# Circulating HPV Tumor DNA and Molecular Residual Disease in HPV-Positive Oropharyngeal Cancers: A Scoping Review

**DOI:** 10.3390/diagnostics14232662

**Published:** 2024-11-26

**Authors:** Andrea Migliorelli, Andrea Ciorba, Marianna Manuelli, Francesco Stomeo, Stefano Pelucchi, Chiara Bianchini

**Affiliations:** ENT & Audiology Unit, Department of Neurosciences, University Hospital of Ferrara, 44100 Ferrara, Italy

**Keywords:** ctHPVDNA, OPSCC, liquid biopsy

## Abstract

The aim of this review is to assess the utility of circulating HPV tumor DNA (ctHPVDNA) clearance in the monitoring of molecular residual disease in HPV-related oropharyngeal squamous cell carcinoma (OPSCC) patients. Recently, ctHPVDNA in patient plasma was found to be a promising biomarker for HPV OPSCC. Changes in this biomarker appear to be associated with treatment response and may be useful for identifying molecular residual disease. A review of the literature was performed using PubMed/MEDLINE, EMBASE, and Cochrane Library databases according to the PRISMA criteria for scoping reviews (from 2017 to July 2024). A total of 5 articles and 562 patients have been included. Three studies examine the role of ctHPVDNA clearance in CRT, while the remaining two studies consider surgery as a treatment option. The results of this scoping review indicate that ctHPVDNA has a potential role to serve as a valuable biomarker in the assessment of molecular residual disease. Further studies are required to confirm the efficacy of this marker for stratifying this group of patients.

## 1. Introduction

Human papillomavirus (HPV) infection is a sexually transmitted disease that is a major cause of numerous cancers, with cervical cancer being the most common. The estimated prevalence of HPV infection among American citizens in 2018 exceeded 80 million, with 14 million new cases being documented in that year [1]. The oncogenic role of HPV in cancer pathogenesis is well established based on studies of cervical carcinoma in women.

HPV represents a large family of undeveloped double-stranded DNA viruses. The virus’s circular genome of approximately 8000 base pairs encodes two structural genes necessary for the assembly of the viral capsid, as well as six non-structural genes (E1, E2, and E4) involved in viral replication and regulation. The remaining three genes, E5, E6, and E7, are associated with HPV-mediated cellular transformation. The E6 and E7 genes represent the most definitive markers as they are the only viral genes that are consistently maintained and expressed in HPV-positive tumor cells [2,3,4,5,6,7,8].

The precise role of HPV infection in the pathogenesis of head and neck squamous cell carcinoma is not clearly defined yet [9]. Head and neck cancers represent the sixth most common form of cancer worldwide. As reported by the Surveillance, Epidemiology, and End Results (SEER) program, there are more than 430,000 new cases of head and neck cancer annually [10]. Over 90% of head and neck cancers are squamous cell carcinomas (HNSCCs) [11]. Patients with HPV-positive oropharyngeal cancers (OPSCCs) exhibit distinct clinical, demographic, and prognostic characteristics compared with those with HPV-negative oropharyngeal cancers. This notable distinction has led to the introduction of a staging differentiation in the eighth edition of the American Joint Committee on Cancer (AJCC) staging system [12]. HPV OPSCCs predominantly affect non-smoking white males and have a significantly superior overall prognosis in comparison with their HPV-negative counterparts. The discrepancy in risk factors, incidence, and prognosis has led to the classification of HPV OPSCC as a distinct entity from HPV-negative OPSCCs [1,12].

HPV OPSCC has a significantly superior prognosis in comparison with HPV-negative OPSCC. The patient population is younger and healthier, which theoretically allows for a longer survival period. Consequently, the impact of treatment on long-term quality of life is more pronounced.

In light of these considerations, the scientific community has recently devoted considerable effort to the implementation of deintensification strategies in therapeutic regimens, with the objective of avoiding overtreatment and enhancing patient quality of life [13,14]. In particular, an increasing number of studies have investigated the potential of liquid biopsy and circulating HPV tumor DNA biomarker (ctHPVDNA) for the diagnosis and follow-up of HPV OPSCC [15,16,17]. The ctHPVDNA is a fragment of the HPV genome and is specific only for patients with HPV-related cancer [18]. Quantitative real-time PCR (q-PCR) is the simplest and most widely used technology to detect HPV DNA; however, in 2020, a kit was approved in the United States that allows for the assay of ctHPVDNA in a standard procedure [19].

The aim of this review is to assess the utility of ctHPVDNA clearance in the monitoring of molecular residual disease in HPV-related OPSCC. To the best of our knowledge, this is the first review analyzing ctHPVDNA accuracy in assessing molecular residual disease in HPV OPSCC patients.

## 2. Materials and Methods

**Figure 1:**
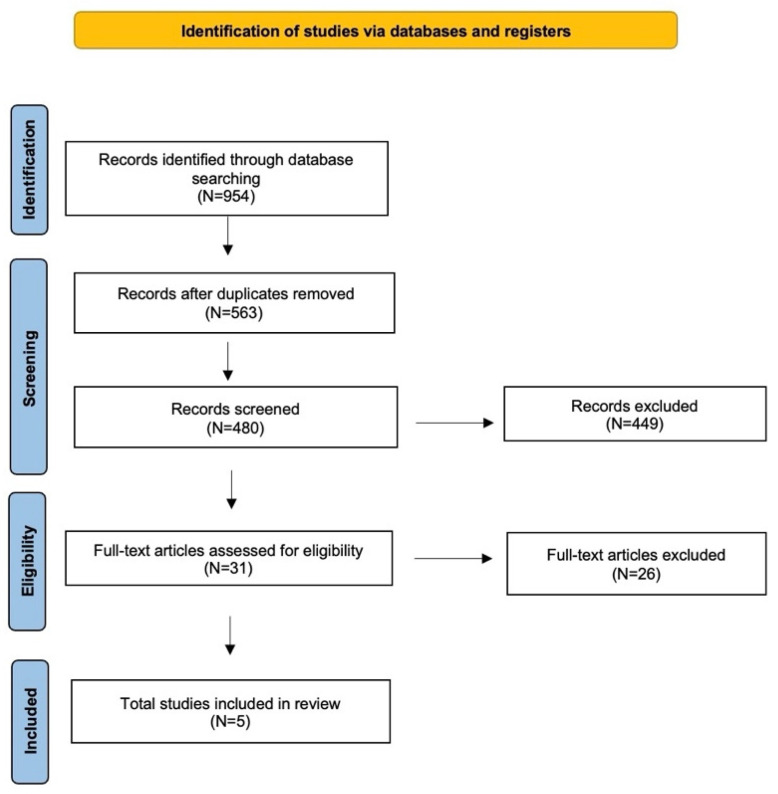
The literature review performed using the PRISMA guidelines for scoping reviews.

A detailed review of the English-language literature on ctHPVDNA in HPV-related OPSCC was performed using PubMed/MEDLINE, EMBASE, and Cochrane Library databases. The search period was from 2017 to July 2024, conducted with the aim of selecting the most recent studies. The terms used were “oropharyngeal cancer”, “HPV related oropharyngeal cancer” or “HPV-OPSCC” and “ctHPVDNA”, “circulating tumor”, “liquid biopsy” or “molecular residual disease”. The search yielded 954 candidate articles. The search was performed according to the “Preferred Reporting Items for Systematic Reviews and Meta-Analyses” (PRISMA) guidelines for scoping reviews (Figure 1) [20]. The inclusion criteria applied were: (i) prospective study; (ii) publication date after 2017; (iii) studies that assessed molecular residual disease by ctHPVDNA in patients with HPV + OPSCC; (iv) clearly defined methods of detection of viral DNA; and (v) English language. Conference abstracts, case reports, retrospective studies, and publications written in a language different from English have been excluded. Two authors (A.M. and M.M.) have independently evaluated all titles, and relevant articles have been individuated according to inclusion/exclusion criteria; a senior author (A.C.) resolved any disagreements. At the end of the full-text review, only 5 articles met the inclusion criteria [21,22,23,24,25,26].

## 3. Results

ctHPVDNA was employed for the assessment of therapeutic efficacy and residual disease in a total of 562 patients. The results of the review process are summarized in Table 1.

The earliest study emerging from the review is that of Chera et al. (2019) [21], in which the authors attempted to perform a risk stratification by studying baseline ctHPVDNA levels and clearance kinetics. The study demonstrated that ctHPVDNA levels at baseline exceeding 200 copies/mL are indicative of genomic tumor biomarkers and, consequently, a more favorable prognosis. Furthermore, patients with a favorable clearance profile, defined as the clearance of more than 95% of the baseline ctHPVDNA values at week four of chemoradiotherapy (CRT), have a more favorable prognosis. In contrast, patients with unfavorable clearance exhibited a higher frequency of disease persistence at the conclusion of therapy.

Subsequently, Cao and colleagues [22] examined ctHPVDNA values in patients with OPSCC p16+ stage III AJCC 8 who had undergone radiochemotherapy. The findings indicate that the alteration in values during the initial two weeks of therapy is a predictor of subsequent therapeutic outcomes. Following a two-week period, the patients were divided into two groups based on whether their ctHPVDNA values had increased or decreased compared with their baseline levels. In particular, a reduction in ctHPVDNA at the two-week mark of CRT in comparison with pre-treatment levels was linked to an increased likelihood of treatment failure or tumor progression within the 12-month period following TR. Furthermore, clearance of ctHPVDNA at weeks 4 or 7 of therapy was not predictive of progression.

In the same year, O’Boyle et al. [23] investigated whether the kinetics of ctHPVDNA clearance following surgical treatment of HPV OPSCC may be associated with the risk of residual disease. The findings were that ctHPVDNA levels below 1 copy/mL on the first postoperative day correlated with no residual disease, patients with levels between 1 and 100 copies/mL with possible microscopic residual disease, and those with levels above 100 copies/mL with macroscopic residual disease.

In 2023, the role of ctHPVDNA at baseline and at the end of treatment was evaluated in patients enrolled in the ARTSCAN III study [24]. The authors discovered that low ctHPVDNA values at the outset of treatment are indicative of a favorable prognostic index for disease-free survival and overall survival. However, no correlation was observed between these values and locoregional control.

In 2024, Souza et al. [25] conducted an evaluation of ctHPVDNA as a tool for the surveillance and assessment of treatment response in patients with HPV OPSCC. The high negative predictive value (98.9% for patients undergoing surgery + adjuvant treatment and 100% for patients undergoing surgery alone) is therefore of importance in the evaluation of definitive treatment. In the event of a positive post-treatment test, further investigation with imaging and closer follow-up is recommended.

Recently, the phase 2 OPTIMAII study, which was conducted in advance-stage patients to evaluate the role of neoadjuvant chemoimmunotherapy, used ctHPV DNA analysis in a subgroup to assess response to neoadjuvant therapy.

Samples were collected from 31 patients before therapy and 6–9 weeks after the start of adjuvant therapy. All patients had detectable levels of HPV DNA at baseline. The 26 patients with complete clearance had a significantly better two-year progression-free survival than those with detectable ctHPV DNA [26].

In summary, all authors have studied the kinetics of ctHPVDNA following treatment. Threshold values are scarcely comparable because of the use of non-standardized measurement methods; at present, it is still not possible to establish an unambiguous cut-off level. However, these studies demonstrate the importance of investigating the kinetics of ctHPVDNA following treatment.

## 4. Discussion

At present, the follow-up protocol for patients with OPSCC is based on imaging and clinical examination. However, the low accuracy and poor diagnostic value of follow-up methods can result in the patient undergoing unnecessary imaging and surgery, leading to overtreatments with a significant negative impact on the patient’s quality of life [27,28,29]. A multitude of deintensification strategies have been the subject of recent studies [30,31,32,33,34,35]. In particular, researchers have investigated the clinical applications of ctHPVDNA in biological fluids for the monitoring of HPV OPSCC patients [15,16,17,36]. The progression of tumors is associated with the expression of oncogenic viral DNA and proteins. It is noteworthy that the circulating EBV DNA load is currently regarded as a novel biomarker that reflects prognosis and changes in response to treatment in nasopharyngeal cancer [37]. Similarly, ctHPVDNA may have a comparable impact and diagnostic efficacy for HPV OPSCC cancers, as proposed by several authors. In fact, a 2023 meta-analysis has demonstrated that droplet digital polymerase chain reaction (ddPCR) for ctDNA has favorable accuracy, sensitivity, and specificity in the diagnosis of HPV-related OPSCC. These authors conducted an analysis of 729 patients with HPV-related OPSCC and ctHPVDNA emerges as a crucial biomarker for diagnosing these patients [16]. Also, another meta-analysis conducted by Campo et al. [15] demonstrated that ctHPVDNA may also be a valuable tool for monitoring patient outcomes; a sensitivity of 86% (95% CI: 78–91%) and a specificity of 96% (95% CI: 91–99%) has been observed in the analysis of 1311 patients with HPV OPSCC.

The present review examines a further feature: the application of ctHPVDNA in the detection of molecular residual disease, thus anticipating findings that can be detected by imaging. To date, there only are a few studies in the literature regarding the use of ctHPVDNA as a biomarker to monitor molecular residual disease, and to the best of our knowledge, this is the first review analyzing the accuracy of ctHPVDNA in assessing molecular residual disease for HPV OPSCC patients. In particular, this scoping review examined the treatment results of 562 patients with HPV-related OPSCC. The mean age of the patients included in the study is consistent, ranging from 60 to 64 years. Three studies have examined the role of ctHPVDNA clearance in CRT, two studies have investigated its role in surgery as a treatment option, and a recent study has considered its role in neoadjuvant chemoimmunotherapy. According to the data of the present review, the positivity rate for liquid biopsy in patients with a confirmed diagnosis of HPV OPSCC via biopsy exhibits considerable variability, with reported rates ranging from 100 to 82%. This can be attributed to the utilization of disparate primers; an increased number of HPV types tested is associated with a heightened probability of obtaining a positive liquid biopsy result, as previously discussed, given that not all HPV-related cancers are determined by the presence of types 16 or 18.

Furthermore, the present review indicates that the timing of sampling for liquid biopsy varies considerably between studies, emphasizing the necessity of a standard protocol. Moreover, the included studies employed disparate kits and primers. The initial studies were conducted to analyze alterations in ctHPVDNA clearance in patients undergoing CRT. The results on the effectiveness of ctHPVDNA clearance in monitoring CRT efficacy and molecular residual disease can be difficult to evaluate. The results of the studies reviewed indicate that the biomarker has significant potential and may become a crucial tool for risk stratification in the future. However, further studies are necessary to establish standardized protocols for its use in clinical practice.

Interestingly, the analysis of ctHPVDNA clearance for monitoring residual disease in patients undergoing surgical treatment could offer significant findings. O’Boyle et al. [23] developed a risk stratification system for macroscopic, microscopic, and nonmolecular residual disease based on the number of copies/mL of ctHPVDNA present as early as the first postoperative day. Consequently, ctHPVDNA levels on the first postoperative day have been correlated with the risk of residual disease. These findings have illustrated the potential utility of ctHPVDNA as a biomarker for personalized treatment in patients with HPV-positive squamous cell carcinoma undergoing surgery.

It is likely that the role of ctHPVDNA in stratifying the risk of molecular residual disease in surgically treated patients is of great importance. At present, the standard post-treatment evaluation is performed with PET at three months; however, this procedure can still retain a high number of false positives and a relatively low positive predictive value of 30% in 12-week surveillance for HPV OPSCC [38,39,40]. Thus, ctHPVDNA could represent a sensitive and effective method for the detection of residual disease. In the near future, it may be used as a complementary technique alongside PET in order to enhance patient follow-up.

Recently, other molecular techniques have been proposed in the literature, aiming to perform targeted follow-up and early detection of residual disease, with a view of enabling personalized and tailored treatments. (i) Some studies have evaluated the presence of HPVDNA in saliva, but these findings, although promising, are still at an early stage, and for the present time, saliva sampling with HPVDNA assay is proposed as a complement to ctHPVDNA and not as its replacement [41,42,43]. Furthermore, recent evidence suggests that it has good potential for assessing treatment response [44]. (ii) MicroRNAs could have the potential to serve as biomarkers for the early detection of patients with residual disease following treatment for HPV OPSCC. Currently, the literature has primarily focused on their use for early detection; however, they may also have a role in the early assessment of treatment response in the future [44]. (iii) Tumor-derived extracellular vesicles (exosomes) are nanometric particles with DNA, RNA, proteins and lipids inside, which are derived from both normal and cancer cells [45]. They have recently been proposed as biomarkers for the evaluation of patients with HPV OPSCC.

In summary, PET is currently the gold standard for assessing response to treatment, but it has many limitations and a high false-positive rate. For the assessment of molecular residual disease, ctHPVDNA represents the most studied and established technique available to date, with promising results. However, micro-RNA and exosome analysis are emerging as possible complementary methods that may improve the results of liquid biopsy by allowing for more personalized treatment of these patients.

ctHPVDNA has demonstrated high specificity and sensitivity in the diagnosis and identification of HPV OPSCC recurrence. The present review has illustrated that ctHPVDNA may be of pivotal importance in surgically treated patients and also following neoadjuvant chemoimmunotherapy. This illustrates the potential role of ctHPVDNA when considering a de-escalation treatment [24]. In our opinion, perioperative ctHPVDNA monitoring will provide useful information for identifying residual disease and for personalizing treatments.

The major drawbacks of this study are (i) the small number of studies available in the literature so far, and therefore those included within this review; (ii) the heterogeneity of the timing of sampling to assess ctHPVDNA clearance; and (iii) the use of different assay kits and primers within the selected studies.

## 5. Conclusions

In conclusion, the results of this scoping review indicate that ctHPVDNA has a potential role to serve as a valuable biomarker in the assessment of molecular residual disease, but the results, although encouraging, are still preliminary.

Data from the literature are poorly comparable due to the different methods and timings of measuring ctHPVDNA, so at present, it is not possible to describe a practical universal procedure (i.e., standard cut-off levels or time of sampling). This technique could eventually be incorporated within the management protocols for patients with HPV OPSCC in the future, aiming (i) to stratify the risk of molecular residual disease and thus (ii) evaluate the most appropriate therapeutic strategy for the treatment of these patients. The objective of this methodology is to minimize overtreatment and improve the quality of life of the patients.

Further prospective, multicenter studies are required to (i) standardize the procedure, (ii) determine the optimal timing for samplings, and (iii) confirm the efficacy of this technique.

## Figures and Tables

**Table 1:** Literature review.

Author (yrs)	Nop	Mean Age	T	N	Treatment	Primers/Probes	Methods	Positive Liquid Biopsy at Diagnosis	Test Timing	Mean Copies/mL Before Treatment (Range)
Chera (2019) [21]	103	60	0: 5 1:15 2: 69 3: 7 4: 7	0: 5 1: 16 2: 82 3: 0	CRT	HPV: 16, 18, 31, 33, 35	ddPCR	92 (89%)	Before treatment Weekly during treatment Each post-treatment follow-up visit	419 copies/mL (8–22,579)
Cao (2022) [22]	34	64	0: 1 3: 3 4: 29	0: 2 1: 28 2: 2 3: 2	CRT	HPV: 16, 18	ddPCR	28 (82%)	Before treatment At weeks 2, 4, and 7 of treatment At 3–6–12 months after the end of treatment	460 copies/mL (0–34,714)
O’Boyle (2022) [23]	49	62	0: 1 1: 23 2: 22 3: 2 4: 1	0: 4 1: 38 2: 3 3: 4	S: 34 CRT: 15	E7 from HPV: 16, 18, 33, 35, 45	ddPCR	48 (98%)	S: pre-treatment; 1 day, 7 days, 30 days, 3 months, and 12 months post-treatment CRT: pre-treatment; weekly during, 3-, and 12-months post-treatment	2076 copies/mL (0–37,350)
Adrian (2023) [24]	136	60	1: 22 2: 60 3: 17 4:37	0: 5 1:10 2:114 3: 7	CRT	Hpv: 6, 11, 16, 18, 11, 16, 18, 26, 30, 31, 33, 35, 39, 40, 42, 43, 45, 51, 52, 53, 54, 56, 58, 59, 61, 62, 66, 67, 68 (a e b), 69, 70, 73, 74, 81, 82, 83, 85, 86, 87, 89, 90, 91, 114	qPCR + Luminex test	124 (91%)	Before and after treatment	67.5 copies/mL
Souza (2024) [25]	240	62.5	0: 44 1/2: 155 3/4: 41	0: 25 1: 171 2: 27 3: 17	S + adjuvant CRT: 60 S: 61 CRT: 100 C: 1 RT: 17	HPV: 16, 18, 31, 33, 35	ddPCR	N/A	Before and after treatment	N/A
Rosenberg (2024) [26]	31	N/A	N/A	N/A	CI Neoadjuvant + S/CT/RT	HPV: 16, 18	HPV-SEQ	31 (100%)	Before and 6–9 weeks after neoadjuvant treatment	N/A

Abbreviation: C: chemotherapy; CI: chemoimmunotherapy; CRT: chemoradiotherapy; ddPCR: digital drop PCR; N: nodes; N/A: not available; Nop: Number of patients; qPCR: quantified by real-time PCR; RT: radiotherapy; S: surgery; SEQ: next-generation sequencing-based safe-sequencing system; T: primary tumor; Yrs: years.

## Data Availability

No new data were created or analyzed in this study.
